# Disinfection of Rooms by Formaldehyde Vapour

**Published:** 1896-08-15

**Authors:** H. S. Wansbrough Jones


### Medical Progress and Hospital Clinics.

[*The Editor will be glad to receive offers of co-operation and contributions from members of the profession. All letters should be addressed to* The Editor, at the Office, 428, Strand, London, W.C.]

### DISINFECTION OF ROOMS BY FORMALDEHYDE VAPOUR.

By H. S. Wansbrough Jones, M.B., C.M.Edin., B.Sc.Lond.

As the short article on this subject in The Hospital for July 25th, has attracted some notice, I propose to further discuss briefly some of the more practical aspects of the subject. It has been asked by one who has had considerable experience of hospital management if "it is thought that the present methods of disinfecting wards and rooms are ineffective." Now I had thought it to be notorious that these methods are ineffective, so did not think it necessary to emphasise the fact. The results of Koch's experiments on the disinfection of rooms were to show that in an air-tight box sulphurous acid in the proportion of 1·0 per cent. of the cubic space killed sensitive bacilli in half an hour, but that anthrax spores resisted 6·0 per cent. for days. A similar result was obtained with chlorine. In ordinary rooms, however, the leakage was so enormous that it was impossible to maintain the theoretical proportion, so that even some of the bacteria themselves escaped. Koch found also that organisms protected from the action of the fumigant—*e.g.*, in pockets, under mattrasses, &c.—were not destroyed by these methods. Though these methods, then, combined with the stripping and washing of walls, and the storing of bedding and clothing have been the best hitherto known, and have been generally employed, they have never been held to be satisfactory. Professor Délepine and Dr. Ransome found sulphurous acid and chlorine vapours useless in disinfecting rooms infected with tubercle bacilli, and recommended washing with bleaching powder. Their experiments showed that this method was effectual in rendering sterile the bacilli and their spores, but their experiments did not extend to other organisms. The limitations of this method also, from the point of view of injury to fabrics and colour, as well as its laboriousness, are only too apparent.

In my first paper I reported experiments by MM. Roux, Trillat, and Bosc, showing that by the use of formaldehyde sterilisation can be made absolutely perfect, and that by simple methods this can be done in an ordinary house or hospital ward, without removing, destroying, or damaging a single article. So powerful is the action of formaldehyde, that it has several times happened in well-known bacteriological laboratories that all the cultures have been killed by inadvertently leaving the stopper out of a ten-ounce bottle of formalin. But, indeed, all experiments on its efficacy have been conclusive in its favour.

It only remains to consider the question of cost, and the removal of the irritating vapour after disinfection has been completed.

As regards the second of these points, Dr. Bosc says that after two hours ventilation no smell remains, even when the doors and windows are closed. In contrast to the simplicity of this method, it must be remembered that to obtain the best results with sulphur or chlorine, not only have the carpets, bedding, fabrics, &c., to be removed and stoved, but also the walls have to be stripped and the floors and paint washed down. Now as to cost. Dr. Bosc's results show that a hospital ward with two side wards opening into it, the total cubic contents of which was roughly 800 cubic metres, could be completely sterilized by 4½ litres of formalin. Translated into English measures this means that 28,253 cubic feet can be disinfected by 10 lbs. of formalin. Now formalin costs 2s. 8d. a pound, so that it would cost £1 6s. 8d. to disinfect this space.

The sulphur necessary for the same space would cost about 8s. (1 lb. sulphur to 1,000 cubic feet).

If chlorine were used according to Koch's directions (15 lbs. chloride of lime and 22 lbs. hydrochloric acid per 1,000 cubic feet) the cost would be £5 11s. But here it must again be noted that, in addition to this there is the cost of washing, stripping, and often repapering, of stoving carpets, bedding, clothes, &c., and of damage to goods injured or destroyed by chlorine or sulphur dioxide.

The original cost of an autoclave large enough for the disinfection of houses or hospitals would be about £8, the cost of the gas used in the operation would be trifling, and the apparatus could be easily managed by anyone of ordinary intelligence; further, it would be unnecessary in hospitals to have a steam disinfector, an apparatus which costs from £150 to £200, to say nothing of the expense of working it.

It would appear then that, in addition to being the only reliable and practical disinfectant for all ordinary purposes, formaldehyde is cheaper and its application simpler.

